# Oncofertility outcomes after fertility-sparing surgery in infertile BOT patients: results of a large retrospective study on controlled ovarian stimulation

**DOI:** 10.3389/fonc.2025.1592600

**Published:** 2025-09-29

**Authors:** Liang Liang, Xiu-Mei Zhen, Yuan Li, Cai-Hong Ma, Shuo Yang, Jie Yan, Xue-Ling Song, Rong Li

**Affiliations:** ^1^ Center for Reproductive Medicine, Department of Obstetrics and Gynecology, Peking University Third Hospital, Beijing, China; ^2^ National Clinical Research Center for Obstetrics and Gynecology (Peking University Third Hospital), Beijing, China; ^3^ State Key Laboratory of Female Fertility Promotion, Department of Obstetrics and Gynecology, Peking University Third Hospital, Beijing, China

**Keywords:** borderline ovarian tumor, fertility-sparing surgery, recurrence, oncofertility, infertility, controlled ovarian stimulation

## Abstract

**Purpose:**

The optimal management of borderline ovarian tumors (BOTs), particularly the safety and efficacy of *in vitro* fertilization (IVF) following fertility-sparing surgery (FSS), remains controversial. This study aimed to evaluate the impact of controlled ovarian stimulation (COS) on oncofertility outcomes in infertile women with BOT after FSS.

**Methods:**

This retrospective observational study was conducted at the Reproductive Medical Center, Peking University Third Hospital (Beijing, China) and included 73 infertile BOT patients who underwent IVF between January 2008 and June 2022.

**Results:**

Median follow-up was 61.0 months (range: 7.0–156.0 months). The BOT recurrence rate was 20.5% (15/73), and the cumulative live birth rate per patient was 57.5% (42/73). There were no significant differences in the number of COS cycles (*P*=0.513) or total dose of gonadotropin (Gn) (*P* =0.183) between the recurrence and non-recurrence groups. Multivariate analysis identified three independent predictors of oncologic outcomes: interval from surgery to first IVF (HR: 0.07; 95% CI: 0.01–0.44; *P* =0.004), duration of Gn administration (HR: 9.70; 95% CI: 1.37–68.70; *P* =0.023), and live birth (HR: 7.02; 95% CI: 1.35–36.41; *P*=0.020).

**Conclusion:**

In infertile BOT patients undergoing FSS, IVF significantly improves pregnancy outcomes. COS can be safely conducted post-FSS without increasing recurrence risk, provided initiation is delayed ≥3 months postoperatively and Gn administration is limited to ≤13 days.

## 1 Introduction

Borderline ovarian tumors (BOTs) are characterized by low-grade malignant potential and infrequently metastasize (1). Approximately 75% of BOTs are diagnosed at stage I, according to the International Federation of Gynecology and Obstetrics (FIGO), and thus carry a favorable prognosis, with 5- and 10-year survival rates of 95% and 90%, respectively (2–5). The mean age at diagnosis is 45 years, but one-third of patients are under 40 and therefore eligible for fertility-sparing surgery (FSS) (6–8). FSS, which involves preserving the uterus and at least part of one ovary, is the standard treatment for young women with BOTs. Although FSS is associated with an increased risk of recurrence (9), most recurrences involve residual ovarian tissue and remain borderline in nature. In such cases, repeat FSS may be performed without compromising overall survival (10).

A meta-analysis reported 54% of women conceive spontaneously following conservative management (11). Notably, 10%–35% of BOT patients exhibit infertility before treatment (12). For those with BOT-associated infertility, assisted reproductive technology (ART) represents a viable option. However, determining the optimal timing for pregnancy remains challenging. Some experts caution against early conception, suggesting that increasing the pelvic blood flow may elevate the risk of recurrence. Consequently, a 2-year delay post-treatment is often recommended (13). Conversely, delayed pregnancy can reduce ovarian reserve, potentially impairing reproductive outcomes. As a result, others advocate for attempting conception as soon as possible after surgery (14). To date, no consensus exists regarding the ideal timing of natural conception or initiation of ART.

Letrozole, an aromatase inhibitor, is used in controlled ovarian stimulation (COS) protocols to minimize estrogen exposure. For BOT patients with postoperative infertility, letrozole alone or combined with low-dose gonadotropin (Gn) may be employed to mitigate the theoretical risk of recurrences. However, these approaches often yield fewer oocytes, fewer available embryos, and lower pregnancy success rates (12, 15, 16). Standard COS procedures using high doses of Gn typically result in satisfactory pregnancy rates, but raise concerns due to elevated circulating estradiol levels, which may adversely affect outcomes in estrogen-sensitive cancers. Nonetheless, data on the oncological safety of these protocols in BOT patients remain limited (17). While some recent case reports suggest that infertility drugs are safe in this population, large-scale studies are lacking (18, 19). This study aims to report the oncologic and fertility outcomes of the largest known BOT cohort to undergo COS.

## 2 Materials and methods

### 2.1 Study design and population

This retrospective observational study included 81 patients with BOT and infertility who underwent IVF or intracytoplasmic sperm injection (ICSI) at the Reproductive Medical Center, Peking University Third Hospital, between January 2008 and June 2022. All patients received FSS. Institutional Review Board approval was obtained (2019SZ-018).

The inclusion criteria were as follows: 1) histopathologically confirmed stage I BOT, 2) diagnosis of infertility, 3) age ≤40 years, 4) completion of FSS and standard COS after surgery, and 5) a minimum of 6 months of follow-up after infertility treatment.

**Figure 1:**
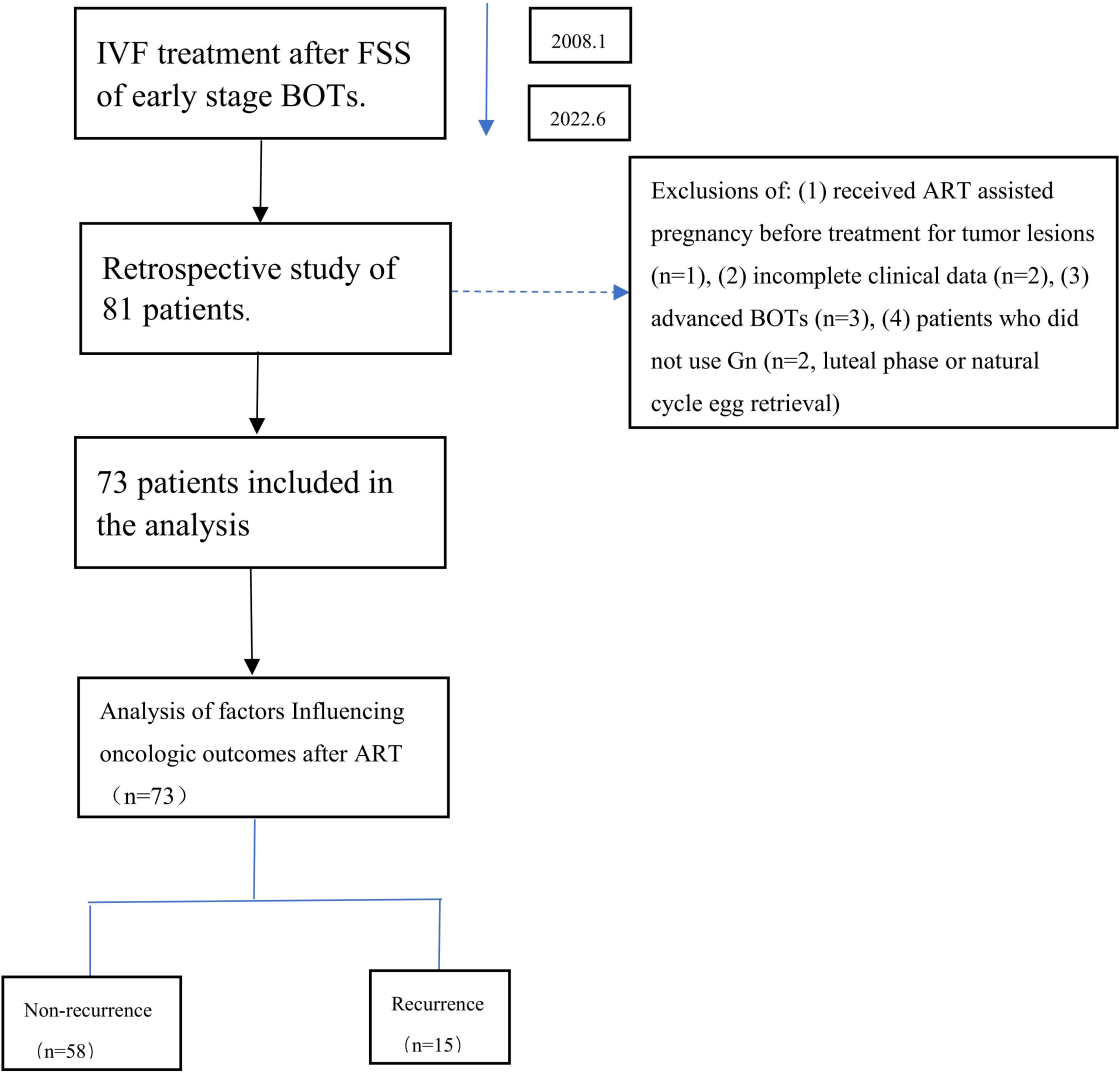
Flowchart of the analysis cohort.

**Figure 1** presents the patient selection flowchart. Of the 81 eligible BOT patients undergoing IVF/ICSI, 73 met the inclusion criteria after exclusions. Clinical data and IVF/ICSI treatment records were extracted from both paper-based and electronic medical records.

### 2.2 Use of fertility-sparing surgery

Patients undergoing conservative treatment were defined as those with preservation of the uterus and at least part of one ovary. Additional surgical staging, when performed, included peritoneal washings, multiple peritoneal biopsies, and omentectomy. The FSS procedures varied depending on tumor laterality; for unilateral BOT, options included cystectomy or unilateral salpingo-oophorectomy (USO); for bilateral BOT, procedures included bilateral cystectomy (BC) or USO with contralateral cystectomy (CC).

### 2.3 Controlled ovarian stimulation protocols

Each patient referred to the Reproductive Medical Center underwent a comprehensive infertility evaluation by reproductive specialists. COS protocols included gonadotropin-releasing hormone agonists (GnRH-a), GnRH antagonists (GnRH-ant), or mild stimulation regimens. Gonadotropins (Gn) used included human menopausal gonadotropin (Lizhu Pharmaceutical Co., China), recombinant follitropin β injection (PUREGON, Merck Sharp & Dohme, Germany), and recombinant human follitropin for injection (GONAL-f, Merck Serono, Germany). Starting doses and adjustments were guided by institutional protocols for both conventional and random-start COS.

#### 2.3.1 Antagonist protocol

COS was initiated on day 2 of a menstrual cycle, with an initial Gn dose of 150–300 IU administered until oocyte triggering. When the dominant follicle reached ≥12 mm in diameter, a daily 0.25-mg GnRH-ant (Cetrotide, Merck Serono, Germany) was added. Final oocyte maturation was triggered using recombinant human chorionic gonadotropin (OVIDREL, Merck Serono S.p.A., Italy) when at least two follicles measured ≥18 mm. Oocyte retrieval was scheduled 36–38 h after triggering.

#### 2.3.2 Agonist protocol

GnRH-a long protocol: Subcutaneous injection of GnRH-a (Triptorelin, IPSEN Pharma Biotech, France) at a dose of 1.25 mg was commenced in the middle luteal phase of the previous menstrual cycle. Gn was administered at 150~300 IU until the trigger day.

GnRH-a short protocol: From menstrual cycle day 2 to day 6, short-acting GnRH-a 0.1 mg/day was injected subcutaneously. Gn was introduced between day 3 and day 5 and continued until the trigger day.

#### 2.3.3 Mild ovarian stimulation protocol

Letrozole (Haizheng Pharmaceutical Co., China) 2.5 mg/day was administered from day 2 of the menstrual cycle for five consecutive days. Gn (75–225 IU) was initiated 2 days after starting letrozole. Once the leading follicle reached ≥14 mm, 0.25 mg/day of the GnRH antagonist was added and continued until the trigger day.

Retrieved oocytes underwent fertilization via conventional IVF or ICSI. Embryos were cultured to day 3 or day 5 post-retrieval. Embryo transfer (ET) timing was guided by institutional policy, favoring day 3 transfer when embryos met quality criteria (at least five to eight cells with 30% maximum fragmentation). Blastocyst morphology was assessed on day 5 using the Gardner scoring system.

### 2.4 Fresh embryo transfer

No more than two day-3 embryos or one day-5 blastocyst were transferred per cycle. Luteal phase support began after oocyte retrieval, using either progesterone sustained-release vaginal gel (Crinone, 90 mg daily; Merck Serono, Switzerland) or dydrogesterone tablets (Duphaston, 20 mg twice daily; Abbott Biologicals B.V., Netherlands). In cases of hydrosalpinx, high risk for ovarian hyperstimulation syndrome, or other contraindications, ET was canceled and all embryos were vitrified. Patients who did not achieve a live birth underwent frozen embryo transfer (FET) cycles until all vitrified embryos were used or a live birth was achieved. Surplus embryos were cryopreserved for future FET attempts.

### 2.5 Frozen-thawed embryo transfer

For patients with regular menstrual cycles, FET was performed in a natural cycle, with cleavage-stage embryos transferred on day 3 post-ovulation and blastocysts on day 5. For patients with irregular or absent menstrual cycles, FET was performed in a hormonally prepared endometrium. Oral estradiol valerate (Progynova, Bayer AG, Germany) was initiated on menstrual cycle day 2 at a standard dosage of 3 mg twice daily. The dose was adjusted according to endometrial thickness. Progesterone was given to transform the endometrium when the endometrium reached ≥8 mm. ET was scheduled according to embryo stage: cleavage-stage embryos after 4 days of progesterone exposure and blastocysts after 6 days.

### 2.6 Follow-up after the end of infertility therapy and recurrence criteria

Patients underwent physical and gynecological examinations, serum CA-125 assessment, and pelvic ultrasound at least every 6 months during the first year post-treatment and annually thereafter. Recurrence was confirmed by surgical intervention and histopathological analysis.

### 2.7 Statistical analysis

Continuous variables with normal distribution were expressed as mean (standard deviation, SD), while categorical data were presented as counts and percentages. Group comparisons for normally distributed variables used *t*-tests; non-normally distributed and categorical data were analyzed using the Mann–Whitney *U* test or Pearson’s *χ*²/Fisher’s exact test as appropriate.

Survival analysis was performed using the Kaplan–Meier with log-rank tests. Multivariate Cox proportional hazards models included variables with *P* ≤0.20 in the univariable and clinically relevant factors.

All tests were two-sided with *α*=0.05. Analyses were performed using SPSS 25.0 (IBM Corp., Armonk, NY, USA).

## 3 Results

### 3.1 Patient characteristics

During the study period, 73 patients were followed up for 7.0–156.0 months, with a median follow-up of 61.0 months. Among these patients, 15 (20.5%) experienced recurrence and 58 (79.5%) remained recurrence-free. Recurrence interval following FSS and COS ranged from 10.0 to 130.0 months (median: 26 months). **Table 1** summarizes the clinical and tumor characteristics of the recurrence group and the non-recurrence group. The mean age of the cohort was 31.4 (3.7) years, with no significant difference between the two groups (*P* =0.697). Of the 73 patients, 36 (49.3%) had serous BOTs, 20 (27.4%) had mucinous BOTs, and 17 (23.3%) exhibited micropapillary features. All patients underwent FSS surgery and were diagnosed at FIGO stage I. Twenty-one patients (28.8%) underwent laparotomy, while 52 (71.2%) underwent laparoscopic surgery. Regarding surgical procedures, 27 patients (37.0%) had unilateral cystectomy (C), 20 (27.4%) had bilateral cystectomy (BC), 11 (15.1%) underwent unilateral salpingo-oophorectomy (USO), and 15 (20.5%) had USO with contralateral cystectomy (CC). There was no significant difference in surgical approach (*P* =0.602), surgical procedures (*P* =0.826), histopathological subtype (*P* =0.757), or prior recurrences before IVF (*P* =0.782) between the recurrence and non-recurrence groups.

**Table 1:** Demographics and surgical findings of patients with conservatively treated BOTs.

Characteristic	Total	Non-recurrence (*n*=58)	Recurrence (*n*=15)	*P*-value
Age (years), mean (SD)	31.4 (3.7)	31.3 (3.9)	31.7 (3.1)	0.697^1^
Duration of infertility years, median (range)	2 (1–13)	2 (1–13)	3 (1–10)	0.129^2^
Type of infertility				0.081^3^
Primary	48 (65.8)	41 (70.7)	7 (46.7)	
Secondary	25 (34.2)	17 (29.3)	8 (53.3)	
Surgical approach				0.602^3^
Laparotomy	21 (28.8)	18 (31.0)	3 (20.0)	
Laparoscopy	52 (71.2)	40 (69.0)	12 (80.0)	
Tumor size				0.490^3^
Unilateral	38 (52.1)	29 (50.0)	9 (60.0)	
Bilateral	35 (47.9)	29 (50.0)	6 (40.0)	
Surgical procedures				0.826^3^
C	27 (37.0)	20 (34.5)	7 (46.7)	
BC	20 (27.4)	17 (29.3)	3 (20.0)	
USO	11 (15.1)	9 (15.5)	2 (13.3)	
USO+CC	15 (20.5)	12 (20.7)	3 (20.0)	
Histology				0.757^3^
Serous	36 (49.3)	28 (48.3)	8 (53.3)	
Mucinous	20 (27.4)	17 (29.3)	3 (20.0)	
Micropapillary	17 (23.3)	13 (22.4)	4 (26.7)	
Number of recurrences prior to IVF, median (range)	0 (0–3)	0 (0–3)	0 (0–2)	0.782^2^

Values are *n* (%), unless stated otherwise.

SD, standard deviation; C, cystectomy; BC, bilateral cystectomy; USO, unilateral salpingo-oophorectomy; CC, contralateral cystectomy; IVF, *in vitro* fertilization.

^1^ *t*-test.

^2^Mann–Whitney *U* test.

^3^ *χ* ^2^ test.

Detailed characteristics of the 15 relapsed patients are presented in **Table 2**. One patient who underwent mild ovarian stimulation progressed to stage IIA ovarian mucinous cystadenocarcinoma. She received complete staging surgery followed by six cycles of chemotherapy and remained disease-free. The remaining 14 patients retained their BOT status without progression or recurrence.

**Table 2:** Demographics and relapse findings of patients with BOTs.

Patient no.	Age (years)	Surgical treatment	Operative approach	Histology	Tumor stage	Number of recurrences prior to IVF	Stage of recurrent tumor	Treatment for recurrence	Recurrence interval	Live birth
1	32	C	Laparotomy	Serous	Ic	2	Ic	C	18	Yes
2	36	C	Laparoscopy	Mucinous	Ic	0	Ic	USO	30	Yes
3	35	C	Laparoscopy	Mucinous	Ia	0	Ic	C	45	No
4	31	C	Laparoscopy	Serous	Ic	0	Ic	USO	34	Yes
5	29	BC	Laparoscopy	Micropapillary	Ic	1	Ic, micropapillary	BC	29	Yes
6	33	USO+C	Laparoscopy	Micropapillary	Ic	0	Ic, micropapillary	USO	22	No
7	30	USO+C	Laparoscopy	Mucinous	Ic	0	I_C_	C	26	Yes
8	32	BC	Laparotomy	Serous	Ia	0	Ic	USO	48	Yes
9	34	C	Laparoscopy	Serous	Ic	0	Ic	USO	29	Yes
10	29	USO	Laparoscopy	Serous	Ia	0	Ic, micropapillary	USO	130	Yes
11	30	C	Laparoscopy	Serous	Ia	0	Ic	C	13	Yes
12	26	C	Laparoscopy	Serous	Ia	0	Ic	USO	12	Yes
13	31	BC	Laparoscopy	Micropapillary	Ic	0	IIa, adenocarcinoma	USO	18	No
14	38	C	Laparotomy	Micropapillary	Ic	1	Ic	BC	18	Yes
15	30	C	Laparoscopy	Serous	Ic	0	Ic	C	10	Yes

C, cystectomy; BC, bilateral cystectomy; USO, unilateral salpingo-oophorectomy; IVF, *in vitro* fertilization.

**Figure 2:**
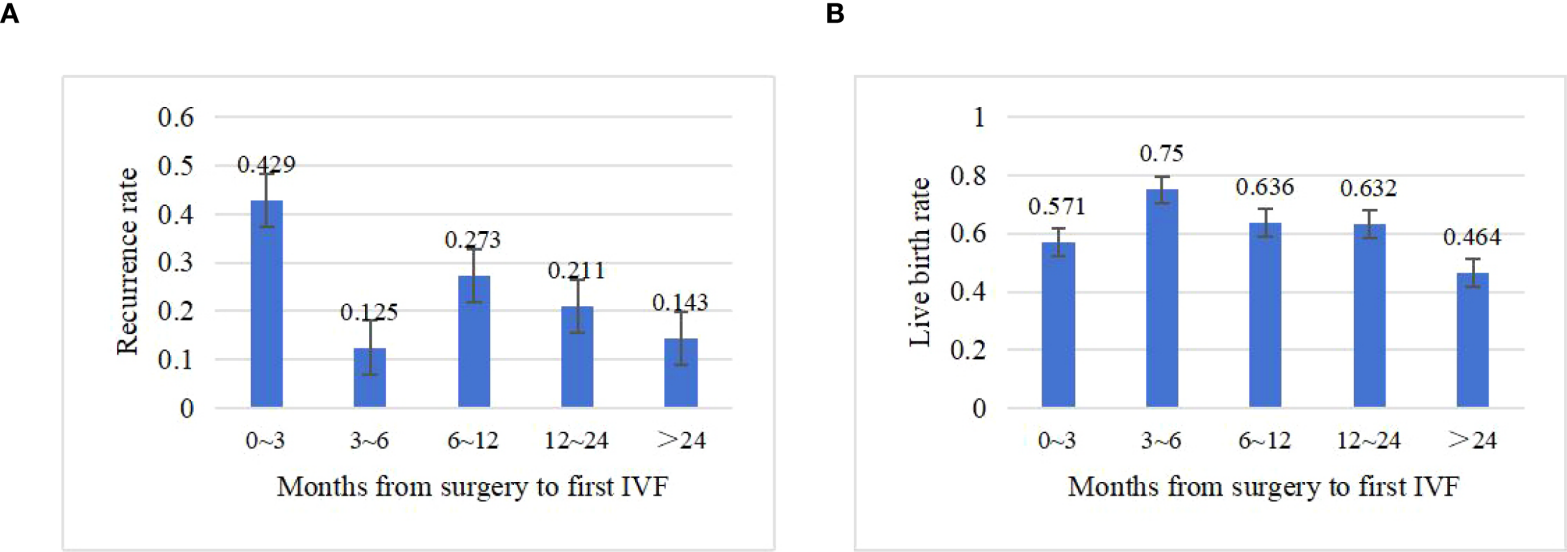
Impact of time to first IVF after surgery on clinical outcomes. **(A)** Recurrence rate by interval (months). **(B)** Live birth rate by interval (months).

**Table 3** summarizes ovarian reserve and COS outcomes after FSS. Basal hormone levels (FSH, LH, E_2_) and AFC showed no significant difference between groups (*P*=0.616, 0.632, 0.584, and 0.794, respectively). The median interval from surgery to first IVF was 20 months (range: 3–146 months), with no significant intergroup difference (21.0 vs. 13.0 months, *P* =0.302). Recurrence rates by interval were as follows: ≤3 months (42.9%), 3–6 months (12.5%), 6–12 months (27.3%), 12–24 months (21.1%), and >24 months (14.3%) (**Figure 2**).

**Table 3:** Ovarian reserve markers and COS outcomes after FSS.

Characteristic	Total	Non-recurrence (*n*=58)	Recurrence (*n*=15)	*P*-value
Interval from surgery to first IVF (range)	20 (3–146)	21 (3–146)	13 (3–72)	0.302^1^
Number of basal AFC, median (range)	7 (2–24)	7 (2–24)	6 (3–24)	0.794^1^
Basal FSH (mIU/mL), mean (SD)	8.0 (3.1)	8.1 (3.4)	7.6 (2.1)	0.616^2^
Basal LH (mIU/mL), mean (SD)	4.4 (2.2)	4.3 (1.9)	4.6 (3.1)	0.632^2^
Basal E_2_ (pmol/L), mean (SD)	176.1 (136.3)	180.6 (150.1)	158.7 (58.5)	0.584^2^
Cycle number median (range)	1 (1–6)	1 (1–5)	1 (1–6)	0.513^1^
Duration of Gn days, median (range)	13 (4–43)	13 (4–43)	12 (7–33)	0.165^1^
Total dose of Gn (IU), median (range)	3,150 (300–13,000)	3,412.5 (300–13,000)	2,062.5 (825–8,437)	0.183^1^
Number of ET cycles, median (SD)	1.6 (1.2)	1.6 (1.2)	1.4 (1.2)	0.452^2^
The cumulative live birth rate, *n* (%)				0.048*^3^
No	31 (42.5)	28 (48.3)	3 (20.0)	
Yes	42 (57.5)	30 (51.7)	12 (80.0)	

AFC, antral follicle count; SD, standard deviation; FSH, follicle-stimulating hormone; LH, luteinizing hormone; E_2_, estradiol; Gn, gonadotropin; ET, embryo transfer.

^1^Mann–Whitney *U* test.

^2^ *t*-test.

^3^ *χ* ^2^ test.

*p<0.05

A total of 123 COS cycles were performed in 73 patients, followed by 69 fresh and 47 frozen embryo transfers (**Table 3**). The median number of COS cycles was 1 (range: 1–6), with an average of 1.6 (1.2) embryo transfers per patient. There were no significant differences in the number of COS cycles between groups (*P*=0.513). The median total dose of Gn was 3,150 IU (range: 300–13,000 IU), and the median duration of Gn was 13 days (range: 4–43 days), with no significant differences (*P*=0.183 and 0.165, respectively). Overall, 42 of 73 patients achieved live births via IVF, resulting in a cumulative clinical pregnancy rate of 57.5% (42/73). Notably, the cumulative live birth rate was significantly higher in the recurrence group (80.0%, 12/15) than in the non-recurrence group (51.7%, 30/58; *P* =0.048).

### 3.2 Univariate and multivariate analyses of oncologic outcomes affecting BOT patients undergoing IVF

**Figure 3:**
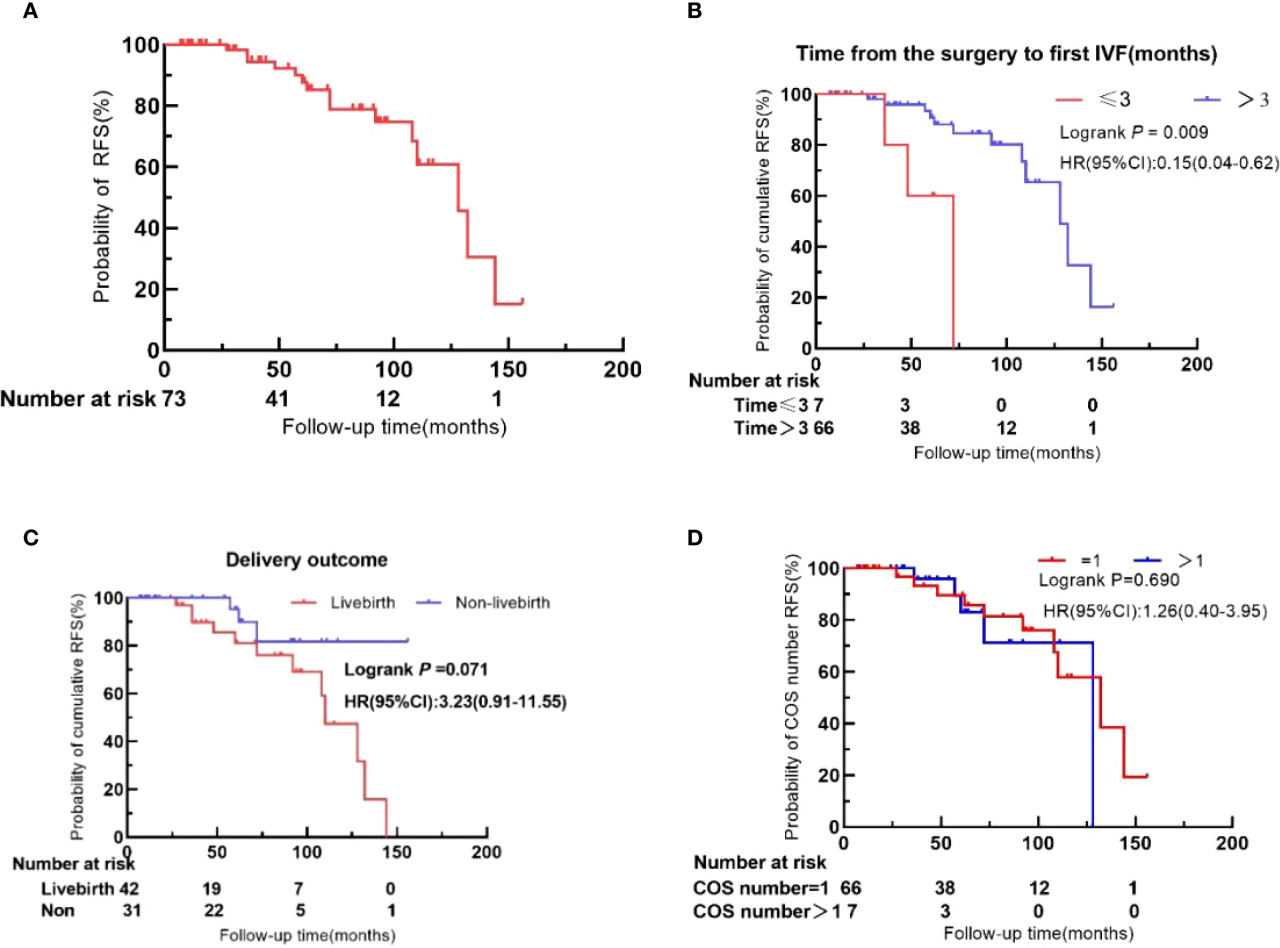
Recurrence-free survival (RFS) in BOT patients after IVF. **(A)** Overall cohort. **(B)** Time to first IVF after surgery (≤3 months vs. >3 months). **(C)** Live birth status. **(D)** Number of COS cycles (1 vs. >1).

Kaplan–Meier survival analysis revealed a 3-year recurrence-free survival (RFS) rate of 94.5% (95% CI: 83.80%–98.18%) and a 5-year RFS rate of 87.7% (95% CI: 74.5%–94.3%) (**Figure 3**).

As detailed in **Table 4**, continuous variables were categorized based on initial patient characteristics, clinical judgment, and relevant literature. Univariate analysis showed that initiating IVF within 3 months after surgery was significantly associated with higher tumor recurrence. In contrast, the number of COS cycles, the number of embryo transfers, basal E_2_ levels, total dose of Gn, and duration of Gn did not significantly influence recurrence.

**Table 4:** Univariate and multivariate Cox regression analysis of factors associated with recurrence.

Variables	Univariate analysis		Multivariate analysis	
	Hazard ratio (95% CI)	*P*-value	Hazard ratio (95% CI)	*P*-value
Age (years)		0.861		
<35	1			
≥35	1.15 (0.25–5.28)			
Histological type		0.504		
Serous	1			
Mucinous	1.23 (0.29–5.14)			
Micropapillary	2.25 (0.60–8.52)			
Duration of infertility (years)		0.622		0.140
<5	1		1	
≥5	0.76 (0.24–2.34)		0.29 (0.05–1.51)	
Type of infertility		0.250		
Primary	1			
Secondary	1.82 (0.66–5.07)			
Basal E_2_ (pmol/L)		0.551		
≤165.0	1			
>165.0	0.72 (0.24–2.13)			
Tumor laterality		0.987		0.947
Unilateral	1		1	
Bilateral	1.01 (0.36–2.86)		0.95 (0.20–4.49)	
Surgical procedures		0.932		0.803
Cystectomy	1		1	
Adnexectomy	1.05 (0.36–3.09)		1.20 (0.29–4.90)	
No. of recurrence prior to IVF		0.227		0.446
0	1		1	
≥1	2.40 (0.65–8.88)		2.03 (0.33–12.55)	
Interval from surgery to first IVF		0.009*		0.004*
≤3	1		1	
>3	0.15 (0.04–0.62)		0.07 (0.01–0.44)	
Total dose of Gn (IU)		0.913		0.078
≤3,150.0	1		1	
>3,150.0	0.94 (0.30–2.91)		0.18 (0.03–1.21)	
Duration of Gn (days)		0.268		0.023*
≤13	1		1	
>13	1.91 (0.62–5.92)		9.70 (1.37–68.70)	
Number of COS cycles		0.690		
≤1	1			
>1	1.26 (0.40–3.95)			
Number of ET cycles		0.484		
≤1	1			
>1	0.68 (0.23–2.04)			
Delivery outcome		0.071		0.020*
No	1		1	
Yes	3.23 (0.91–11.55)		7.02 (1.35–36.41)	

E_2_, estradiol; IVF, *in vitro* fertilization; Gn, gonadotropin; COS, controlled ovarian stimulation; ET, embryo transfer.

*p<0.05

Based on univariate Cox analysis, clinical experience, and supporting literature, eight variables were selected for multivariate Cox regression analysis: years of infertility, tumor laterality, surgical procedures, number of recurrences prior to IVF, interval from surgery to first IVF, duration of Gn, total dose of Gn, and live birth. The analysis identified three independent prognostic factors for oncologic outcomes: interval from surgery to first IVF (HR: 0.07; 95% CI: 0.01–0.44; *P* =0.004), duration of Gn (HR: 9.70; 95% CI: 1.37–68.70; *P* =0.023), and live birth (HR: 7.02; 95% CI: 1.35–36.41; *P* =0.020).

## 4 Discussion

In this single-center retrospective study of 73 BOT patients, IVF following FSS yielded favorable reproductive outcomes without increased oncological risk. The cumulative live birth rate was 57.5% (42/73), indicating that COS—when appropriately timed and tailored in terms of Gn dosage and duration—does not compromise oncologic safety in BOT patients after FSS. FSS involves preserving the uterus and at least part of one ovary. BOT cells have been detected in cryopreserved ovarian tissue, suggesting the potential presence of microscopic disease even in ovaries deemed macroscopically normal (20). Reported recurrence rates following FSS range from 10% to 35% (7, 10, 11, 13), which is higher than the 0%–7.7% recurrence rate observed with radical surgery (21, 22). In the COS process, the circulating estradiol is elevated due to the development of multiple large follicles at once that may worsen oncological outcome in estrogen-sensitive cancers (17). However, Basille et al. demonstrated that neither FSH nor E_2_ promoted the proliferation of cultured BOT cells, despite the presence of their receptors (23). Daraï et al. reported a 27% recurrence rate among 105 patients who received infertility treatments after conservative surgery (11), while Denschlag et al. found a 19% recurrence rate in a systematic review of 62 BOT women who were exposed to COS after FSS (24). This study observed a 20.5% recurrence rate, aligning with established recurrence risks for FSS alone and suggesting that COS does not significantly elevate recurrence risk. In conclusion, available evidence, including our findings, supports the oncologic safety of COS in BOT patients after FSS.

Undergoing at least one COS cycle entails repeated exposure to supraphysiologic estrogen levels, heightened ovarian cell proliferation, and multiple oocyte retrievals. Repeated puncture of the ovaries during oocyte retrieval leads to ongoing tissue injury and repair, theoretically increasing the risk of malignant transformation of ovarian epithelial cells (25). However, our data demonstrate no significant association between the number of COS cycles and BOT recurrence. Patients undergoing ≥2 COS cycles had recurrence rates comparable to those with a single cycle (HR: 1.26; 95% CI: 0.40–3.95; *P* =0.690). These findings suggest that repeated COS does not cumulatively increase recurrence risk. This represents the first clinical evidence to quantitatively assess recurrence risk based on COS cycle frequency in BOT patients. Larger, multicenter studies are warranted to validate these results.

Gn plays a central role in COS by stimulating dominant follicle development and increasing oocyte yield. Variability in COS regimens—including differences in timing and protocol—manifests in divergent Gn dosage and treatment durations, subsequently influencing serum E_2_ level on the trigger day. Currently, there is a lack of data correlating Gn dosage or duration with BOT recurrence. In our study, duration of Gn >13 days emerged as an independent predictor of recurrence (HR: 9.70; 95% CI: 1.37–68.70; *P*=0.023), while total dosage of Gn showed no significant association with recurrence risk. Suboptimal or absent Gn administration impairs ovarian response and oocyte yield, whereas higher Gn dosages increase follicular recruitment and estrogen production—factors potentially contributing to tumor recurrence. Thus, individualized COS planning is essential. Clinicians should tailor Gn dosing based on ovarian reserve, monitor follicular development, and limit Gn duration to ≤13 days to mitigate excessive exposure and enhance both safety and success rates. This strategy optimizes oocyte yield while minimizing oncologic risk.

BOTs are characterized by low malignant potential, and routine postoperative chemotherapy is not recommended by the NCCN guidelines (26). Although there is no urgent time constraint for initiating COS, optimal pregnancy timing remains a critical consideration. Trope et al. proposed delaying pregnancy for 1–2 years postoperatively to reduce early recurrence (27). However, among BOT patients undergoing FSS, 79% experience at least one recurrence, with reported intervals as short as 3 months and a mean recurrence time of 24.0 ± 14.0 months, particularly in bilateral BOTs (28, 29). Repeated ovarian surgeries further diminish healthy ovarian tissue, increase the risk of adhesions, and compromise future fertility (21). Sobiczewski et al. acknowledged the challenge of pinpointing an ideal conception time but advised that patients should not delay pregnancy attempts, recommending resumption of sexual activity once recovery is complete (14). A history of infertility correlates with reduced post-FSS pregnancy rates (30), likely due to persistent underlying conditions or surgical consequences such as adhesions or impaired ovarian reserve. To date, no clear consensus exists on when to initiate ART after FSS in BOT patients. Our study showed recurrence rates of 42.9%, 12.5%, 27.3%, 21.1%, and 14.3% for intervals of ≤3 months, 3–6 months, 6–12 months, 12–24 months, and >24 months, respectively, between surgery and the first COS cycle. Notably, COS initiated within 3 months postoperatively was associated with recurrence rates markedly higher than those reported in the literature for BOTs after FSS. Multivariate Cox analysis further identified initiation of IVF within 3 months of surgery as an independent risk factor for recurrence (HR: 0.07; 95% CI: 0.01–0.44; *P* =0.004). Therefore, COS should be avoided within the first 3 months after surgery. Our data suggest that initiating COS 3–6 months after surgery may provide a lower recurrence risk and higher live birth rate, though the underlying mechanism remains unclear. In a retrospective study, menstrual resumption post-FSS ranged from 1 to 5 months (31), and the median time to conception in patients undergoing laparoscopic surgery for unexplained infertility was 5 months (range: 1–26 months) (32). These observations imply that improved outcomes during the 3–6-month interval may relate to recovery of ovarian endocrine function and resolution of pelvic pathology. Based on these findings, we recommend delaying COS initiation until at least 3 months post-FSS, with particular emphasis on the 3–6-month window as a potentially optimal timeframe for achieving successful and safe pregnancy outcomes. Nevertheless, given the limited sample size in our study, stratified analysis was not feasible. Further research with larger cohorts is essential to confirm these findings.

A key and somewhat unexpected finding of our study is the significantly higher live birth rate observed in patients who experienced disease recurrence compared to those who remained recurrence-free (HR: 7.02; 95% CI: 1.35–36.41; *P* =0.020). We hypothesize that this association may be mechanistically linked to increased pelvic blood flow during pregnancy, potentially altering the tumor microenvironment (27). This finding underscores the importance of incorporating pregnancy history into prognostic evaluation and clinical management. Additionally, the impact of exogenous progesterone exposure warrants careful consideration. A nationwide Danish cohort study by Bjørnhol et al. reported an elevated risk of serous BOTs associated with progesterone use (RR: 1.82; 95% CI: 1.03–3.24), particularly with more than four cycles of use (RR: 2.63; 95% CI: 1.04–6.64). The authors suggested that the elevated expression of progesterone receptors in serous ovarian tumors, as compared to mucinous types, may underlie this increased susceptibility (33). Given that exogenous progesterone is routinely administered during luteal phase support in IVF protocols, initiated on the day of oocyte retrieval and continued through 8–10 weeks of gestation, its potential impact on the BOT recurrence risk merits rigorous future investigation.

This study is constrained by the rarity of BOT and the low recurrence rates. Key methodological limitations include 1) suboptimal statistical power despite the inclusion of the maximum feasible sample size; 2) a limited cohort of BOT patients undergoing FSS with subsequent COS, precluding comprehensive subgroup analyses; and 3) potential institutional bias inherent to this retrospective single-center design. Nevertheless, as a retrospective single-center study, key strengths include the use of standardized COS protocols under consistent clinical and laboratory oversight, which minimized procedural heterogeneity, along with a median follow-up period of 61 months. These methodological features strengthen the clinical relevance and reliability of our guidance for oncofertility practice. Future multicenter studies are essential to validate these clinical recommendations.

## 5 Conclusion

Our results demonstrate that COS can be safely performed in BOT patients following FSS without increasing recurrence risk. Based on current evidence, we recommend initiating COS no earlier than 3 months post-FSS, prioritizing within the 3–6-month window. We also advise limiting Gn duration to ≤13 days and carefully evaluating luteal support regimens, especially in patients with serous BOTs. Future multicenter prospective studies are warranted to validate these clinical recommendations and to further elucidate underlying molecular mechanisms.

## Data Availability

The raw data supporting the conclusions of this article will be made available by the authors, without undue reservation.
